# A Western Case of Intravascular Large B-Cell Lymphoma as Unusual Cause of Persistent Fever

**DOI:** 10.1155/2019/1480710

**Published:** 2019-11-16

**Authors:** Pier Paolo Piccaluga, Stefania Paolini, Cristina Campidelli, Nicola Vianelli, Luigi Bolondi

**Affiliations:** ^1^Department of Experimental, Diagnostic and Speciality Medicine, University of Bologna, Bologna, Italy; ^2^Istituto Euro-Mediterraneo di Scienza e Tecnologia (IEMEST), Palermo, Italy; ^3^Jomo Kenyatta University of Agriculture and Technology, Juja, Kenya; ^4^S. Orsola-Malpighi Hospital, Hematology and Hematopathology Units, Bologna, Italy; ^5^Azienda Ospedaliera Ospedali di Legnano, U. O. Anatomia Patologica, Legnao, Milan, Italy; ^6^Department of Medical and Surgical Sciences, University of Bologna, Bologna, Italy

## Abstract

Fever of unknown origin (FUO) is a common and challenging clinical condition that can be referred, among others, to infections, drug's effects, various inflammatory disorders, and cancers. Among the latter, lymphomas can indeed cause fever, which is therefore accounted as a lymphoma-related sign in patients' staging. Intravascular large B-cell lymphoma (IVLBCL) is a very rare tumor, characterized by lymphoma cell accumulation within sinusoids and, despite a very aggressive course, the evidence of this disease is scarce. Two variants are currently recognized, respectively, occurring in either Western (mainly characterized by neurological symptoms and skin involvement) or Eastern countries (with hemophagocytic syndrome, bone marrow, spleen, and liver involvement). We describe an atypical and unprecedented IVLBCL patient, presenting with pronounced features of Eastern cases as well as skin involvement. Due to the scant amount of neoplastic cells, the diagnosis was very challenging, with FUO being the first and for a certain time unique clinical sign. Although lymphoma was suspected, the lack of evidence for neoplastic cells delayed the final diagnosis. Eventually, only autopsy revealed the extensive involvement of different organs and tissues.

## 1. Background

Fever of unknown origin (FUO) is a relatively common medical condition, more often related to unrecognized infections, autoimmune diseases, drug administration, and cancer [1]. As far as the latter is concerned, fever can represent the initial symptom at presentation. In particular, lymphomas are often associated with fever or other systemic symptoms, their presence defining a more advanced disease [2]. Intravascular large B-cell lymphoma (IVLBCL) is a rare, clinically aggressive lymphoma characterized by an almost exclusive growth of large cells within the lumen of all sized blood vessels [3]. Clinically, in its classical variant, IVLBCL presents with many nonspecific signs and symptoms such as FUO and involvement of the central nervous system (CNS) and skin [3]. The so-called cutaneous variant, in which only the skin is affected, has been more often described in Western countries and has a better prognosis. On the contrary, a hemophagocytic variant associated with hemophagocytic syndrome and often with hepatosplenic involvement and cytopenia can be encountered, more often in Asia [3]. The generally very poor prognosis of this disease has been substantially improved by immunochemotherapy, in particular with anti-CD20 monoclonal antibodies [3]. However, the majority of patients still experience relapse, especially when the CNS is involved. Furthermore, due to the puzzling presentation, the diagnosis is often delayed and not infrequently the disease is recognized *postmortem*.

We describe a patient who presented with persistent fever and died, unfortunately, before a conclusive diagnosis was made.

## 2. Case Report

A 58-year-old Caucasian woman was admitted to our hospital as a case of fever of unknown origin (FUO) after 2 weeks of continuous remittent fever without shivering, polyarthralgias, cervical pain, and headache. On admission, physical examination revealed oedema of the lower limbs with erythematous and painful skin and a temperature of 38°C, not responding to paracetamol and cephalosporin therapy. Laboratory investigations showed an erythrocyte sedimentation rate (ESR) of 120 mm/h, a C-reactive protein (CRP) level of 225 mg/L, a serum ferritin level of 2076 mg/L, and a fibrinogen level of 223 mg/dL. Liver enzymes (AST 83 U/L and ALT 114 U/L) and lactate dehydrogenase (LDH 746 U/L) levels were increased, with reduction of serum albumin and total plasmatic protein values (2.7 g/dL and 5.5** **g/dL, respectively). Procalcitonin was not tested at this time, not being sensitive enough, in our opinion, to discriminate, in this case between infection and malignancy. All inflammatory markers, in fact, were altered.

Acute infectious diseases (hepatitis A, hepatitis B, hepatitis C, and hepatitis E virus, human immunodeficiency virus, cytomegalovirus, parvovirus B19, Epstein–Barr virus, echoviruses, poliovirus, coxsackieviruses, *Treponema pallidum*, *Coxiella burnetii*, *Toxoplasma*, *Salmonella typhi*, *Legionella pneumophila*, *Mycoplasma pneumoniae*, *Leishmania donovani*, *Chlamydya*, *Brucella*, *Leptospira*, *Rickettsia*, and *Borrelia* species IgM) were negative. In addition, Epstein–Barr virus, herpes zoster, herpes simplex, and *Cytomegalovirus* IgG were detected, revealing a previous exposition. Increased *B. burgdorferi* IgG titer was detected by indirect immunofluorescence assay (IIF), but this finding was not confirmed by western blot (WB) technique. Antistreptolysin-O titers results did not recover beta-haemolytic streptococcal infection. Nasopharyngeal and rectal swab results were negative for aerobic bacteria, pseudomonas, and fungi, while *Candida albicans* and *Candida glabrata* were isolated from oral cavity and vaginal swabs. Peripheral blood smears showed no evidence supporting malarial infection. Mantoux intradermic test failed to demonstrate an immune reaction against mycobacterial species. Bacterial (*Salmonella*, *Shigella*, and C*ampylobacter*), mycobacterial, and parasitic culture obtained from blood, stool, and urinary samples were negative. Cerebrospinal fluid examination was performed, but again viral and bacterial involvement could not be proved. Similarly, galactomannan test was negative.

Serum protein electrophoresis presented a slight enlargement of the gamma zone; urine protein electrophoresis disclosed a nonspecific glomerular proteinuria. Immunoglobulin levels (IgG 763 mg/dL, IgA 319 mg/dL, and IgM 316 mg/dL), peripheral T- and B-cell subsets (CD4+CD3+ 59%, CD8+CD3+ 18%, CD3+ 77%, CD19+ 19%, CD16+CD56+ 3%, and CD4+CD8+ 0%), and autoantibodies were regular.

Transoesophageal echocardiography ruled out endocardial vegetations. Abdominal US scan displayed mild hepatomegaly and normal spleen diameters. Whole body computed tomography (CT), single-photon emission computed tomography with labelled leukocytes, bone and lung scintigraphy, and 18 (F)-fluorodeoxyglucose (FDG)-PET were normal. Cerebral magnetic resonance imaging was consistent with a nonspecific subacute inflammatory condition.

Esophagogastroduodenoscopy with duodenal biopsy was not conclusive for Whipple disease. Liver biopsy showed mild piecemeal necrosis with lobular spotty necrosis and cholestatic changes. Bone marrow trephine biopsy was hypocellular with a mild increase in granulopoietic and erythropoietic precursors and reactive plasmacytosis, and no evidence of leishmanial infection.

During the hospitalization, the patient did not respond to ciprofloxacin, imipenem, prednisone (initiated after admission to control fever and symptoms), and NSAID administration, maintaining an acceptable cenesthesis with an unchanged fever curve and persistence of the lower limb lesions. Subsequently, the patient developed an increasing normocytic anemia (RBC 2.51 × 10^6^/*μ*L, HGB 7.9 g/dL, HCT 20.8%, MCV 82.8 fL, MCH 31.5 pg, MCHC 38.0%, RDW 15.2%, and HDW 3.6 g/dL), with hypoalbuminemia (2.5 g/dL) and rising values of acute-phase proteins (CRP 38.6 mg/dL and fibrinogen 920 mg/dL) and liver enzymes: LDH (1131 U/L), alkaline phosphatase (617 U/L), bilirubin (11.87 mg/dL), and transaminase (ALT 129 U/L and AST 64 U/L). An empirical treatment with doxycyclin and AmBisome was commenced on suspicion of chlamydial or leishmania infection, without improvement.

Subsequently, the patient presented a worsening with intermittent delirant states, dyspnea with hypoxia, and thrombotic microangiopathy (TMA) associated with severe thrombocytopenia (3 × 10^9^/L). A second bone marrow trephine biopsy showed a mildly decreased hematopoiesis with a pronounced plasmocytosis and evidence of hemophagocytosis. Prednisone dose was increased (80 mg per day), and a plasma exchange treatment was started. By contrast, etoposide therapy was not initiated due to the suspect of cancer, in order not to compromise the following treatment. After a brief period of symptom improvement, the patient exhibited acute renal impairment, hyperbilirubinemia (18.26 mg/dL), respiratory failure with tachypnea, and severe metabolic acidosis. She was transferred to the intensive care unit (ICU) and developed progressive multiorgan failure, mental cloudiness, and massive nasogastric bleeding; afterwards, she quickly deteriorated and died.

**Figure 1:**
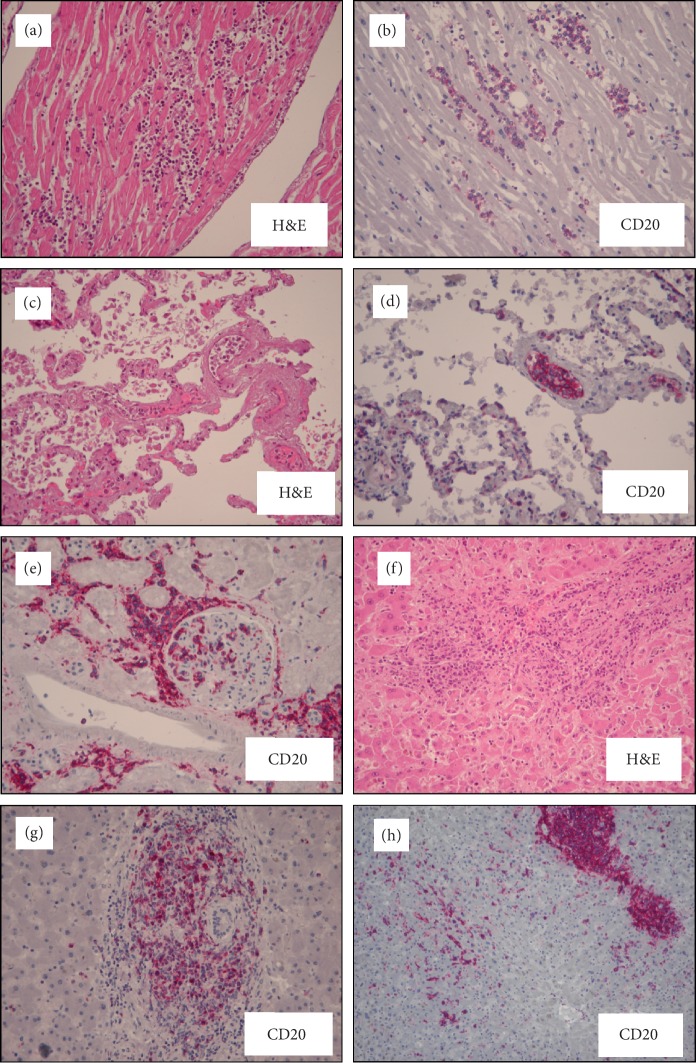
Microscopic appearance of IVLBCL infiltrating heart (a, b), lung (c, d), kidney (e), and liver (f and g) portal localization; (h) sinusoidal localization). H&E = hematoxylin and eosin.

**Figure 2:**
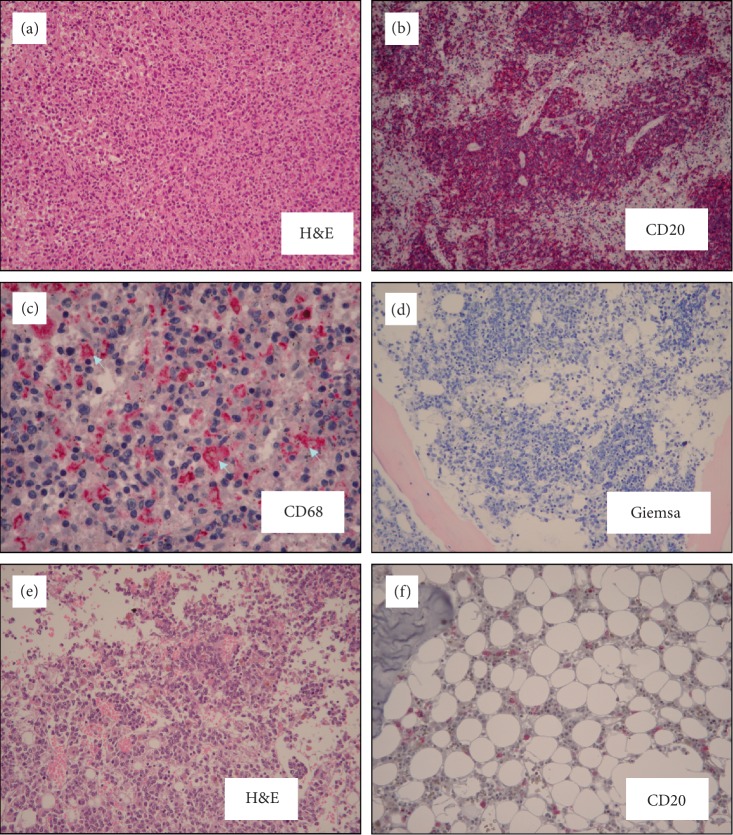
Microscopic appearance of IVLBCL infiltrating the spleen (a–c) and the bone marrow (d–f). Hemophagocytosis (light blue arrows) was observed in the spleen (c). H&E = hematoxylin and eosin.

Autopsy disclosed diffuse involvement by intravascular large B-cell lymphoma (IVLBCL) in several organs (heart, lungs, stomach, kidneys, adrenal glands, liver, and spleen). Multiple skin sampling revealed the presence of rare intraluminal neoplastic elements. Bone marrow specimens were massively infiltrated by neoplastic cells. Furthermore, the liver and spleen showed presence of hemophagocytosis (Figures 1 and 2). Immunophenotyping indicated that neoplastic cells were CD45+, CD20+, CD79a+, BCL2+/− (10–20% of the neoplastic population), CD3−, CD10−, CD5−, and BCL6−, with high Ki67 index (60%).

## 3. Discussion

Intravascular large B-cell lymphoma is an infrequent subtype of diffuse large B-cell lymphoma characterized by the selective growth of neoplastic cells within the lumina of small- to medium-sized blood vessels [2]. This condition was first described in 1959 by Pfleger and Tappeiner as angioendotheliomatosis proliferans systemisata [4]; only during the 1980s, the lymphoid nature of the disease was established [5–8]. IVLBCL has an aggressive behaviour with a poor outcome, and in many instances it is diagnosed postmortem; clinical presentation is extremely variable: B symptoms are the overriding presenting feature, and frequently it can manifest as FUO, which is more commonly reported than in other extranodal aggressive lymphomas (45% vs. 25% of cases at presentation) [9, 10].

Different clinical manifestations have been described depending on geographical origin [8, 9]. Interestingly, our patient showed features typical both of Asian series (HPS, fever, anemia with thrombocytopenia, and liver and bone marrow involvement) and of Western ones (neurological symptoms and skin involvement). In particular, HPS is the most frequent clinical manifestation of Asian-variant IVLBCL, while it is uncommon in European series [11, 12]. Ferreri and colleagues [12] suggested that differences in clinical-pathological features in IVLBCL might be more related to the co-occurrence of HPS rather than the geographical distribution of the disease; in fact, they reported that non-Japanese patients with IVLBCL and HPS showed features similar to those of Japanese HPS patients, while Japanese patients with IVL without HPS had a clinical picture similar to Western cases. Among all Western series of IVLBCL, histologically confirmed HPS has been demonstrated in only five cases [13–16] and two of them were of Caribbean and Vietnamese origin [12]. Our case, as other rare cases of Western HPS-associated IVLBCL, displayed significantly higher rates of advanced disease, fever, hepatosplenic involvement, bone marrow infiltration, fatigue, dyspnea, anemia, thrombocytopenia, and altered liver function tests. This admixed clinical picture probably contributed to mislead the diagnostic process. Unfortunately, it is impossible to assess whether an empiric treatment with etoposide, aimed to reduce HPS, might have allowed a more effective diagnosis.

Neurological disorders are present at initial diagnosis in 34% of European patients [17]; symptoms are miscellaneous, comprising alteration of conscience, peripheral nerves deficit, seizures, paresis, dementia, and extrapiramidal symptoms. Neuroimaging features are nonspecific: CT and MRI can mimic vasculitis, small-vessel ischemic disease, or demyelination [18]. In view of these considerations, suspecting IVLBCL on the basis of neurological symptoms is difficult; moreover, our patient developed delirant states only in the last part of the hospitalization. Skin lesions may present as erythematous eruption, plaques, cellulitis, ulcerated nodules, and, rarely, generalised telangiectasia [9]. Laboratory tests are altered in all cases of IVLBCL: the most represented is increased concentration of serum LDH, followed by hypoalbuminemia (<36 g/L), hyperbilirubinemia (>15 mg/L), hypercreatininemia (>15 mg/L), and increased C-reactive protein (>50 mg/L). Anemia is the most commonly observed blood cell count abnormality, followed by leukopenia and thrombocytopenia [19].

Up to now, no standard procedure for IVLBCL diagnosis has been established. Organ biopsy seems to be the most accurate diagnostic tool, but its importance strictly depends on the choice of the correct biopsy site. Several reports recommend random skin biopsies and repeated bone marrow biopsies in order to obtain a prompt diagnosis [19–24]. Conventional staging investigations (neuroimaging and total-body computed tomography and _18_ (F)-FDG-positron emission tomography) show lower accuracy compared to conventional DLBCL in detecting the presence and the extent of the disease [25–27].

Mourad and colleagues [28] proposed a comprehensive algorithm for an approach to FUO (including abdominal CT, Tc-based nuclear scan, investigation for infective endocarditis, leg doppler imaging, and liver biopsy), with particular emphasis on the role of abdominal CT and nuclear imaging in detecting lymphoproliferative disorders; nevertheless, results may be negative in patients with IVLBCL.

Bone marrow evaluation is a standard procedure in patients affected by FUO. In this case, two bone marrow biopsies were performed, a hematological disease being suspected, but both were not conclusive; only postmortem revealed a massive lymphomatous involvement.

Last of all, the possibility of IVLBCL should be considered in unexplained reactive haemophagocytic syndrome and, even if the bone marrow is infrequently involved. Bone marrow biopsy seems to be the most useful tool when the clinical presentation is characterized by fever of unknown origin with anemia or thrombocytopenia [29]. In this regard, the recent advent of liquid biopsy might represent an attractive strategy [30].

The present case represents, to the best of our knowledge, the first example of IVLBCL, Asian variant, occurring in a European patient. Intensive diagnosis approaches are recommended in patients presenting with FUO, IVLBCL being in the spectrum of differential diagnosis.
